# Aerosol Inhalation of Luteolin-7-O-Glucuronide Exerts Anti-Inflammatory Effects by Inhibiting NLRP3 Inflammasome Activation

**DOI:** 10.3390/ph17121731

**Published:** 2024-12-21

**Authors:** Jianliang Li, Ling Song, Han Li, Yunhang Gao, Tengfei Chen, Zhongxiu Zhang, Hongping Hou, Zuguang Ye, Guangping Zhang

**Affiliations:** Institute of Chinese Materia Medica, China Academy of Chinese Medical Sciences, No. 16, Nanxiao Street, Dongzhimen, Dongcheng District, Beijing 100700, China; jianliang0320@126.com (J.L.); songling1@126.com (L.S.); wzf38@126.com (H.L.); gaoyunhangyhg@163.com (Y.G.); tfchen@icmm.ac.cn (T.C.); zxzhang@icmm.ac.cn (Z.Z.); hphou@icmm.ac.cn (H.H.)

**Keywords:** acute lung injury, aerosol delivery, luteolin-7-O-glucuronide, Inflammation inhibition, NLRP3 inflammasome

## Abstract

**Background:** Luteolin-7-O-glucuronide (L7Gn) is a flavonoid isolated from numerous traditional Chinese herbal medicines that exerts anti-inflammatory effects. Previous research has revealed that aerosol inhalation is the most straightforward way of administration for the delivery of respiratory agents. Thus far, the impact of aerosol inhalation of L7Gn on lung inflammation and the underlying mechanisms remain unknown. **Methods:** The real-time particle size for L7Gn aerosol inhalation was detected by the Spraytec spray droplet size measurement system, including transmission and size diameters. The acute lung injury (ALI) rat model was induced by aerosol inhalation of LPS to evaluate the protective effect of L7Gn. The inhibitory effect of NLRP3 inflammasome activation assays was conducted in LPS-induced MH-S cells. Elisa, Western blotting, and RT-PCR were utilized to investigate the expression of NLRP3 inflammasome-relevant proteins and genes. **Results:** In this study, we found that inhalation of L7Gn aerosol significantly reduced pulmonary injury by inhibiting inflammatory infiltration and enhancing lung function. Meanwhile, the NLR family pyrin domain containing 3 (NLRP3) inflammasome was activated dramatically, accompanied by upregulated expression of IL-1β and IL-18, both in the ALI rat model and in LPS-induced MH-S cells. Moreover, L7Gn was found to significantly downregulate the expression of NLRP3, ASC, caspase-1, and cleaved caspase-1, which are critical components of the NLRP3 inflammasome, as well as the expression of IL-1β and IL-18. **Conclusions:** Based on our findings, L7Gn could exert anti-inflammatory effects by inhibiting NLRP3 inflammasome activation, which may emerge as potential therapeutic agents for the treatment of ALI.

## 1. Introduction

Acute lung injury (ALI) or acute respiratory distress syndrome (ARDS) is a critical respiratory illness that manifests as rapidly progressive dyspnea, tachypnea, and hypoxemia [1,2]. In addition, ALI has been recognized as a severe inflammatory reaction induced by respiratory viruses, bacteria, and physical trauma [3]. Lipopolysaccharide (LPS), a major component of Gram-negative bacterial cell walls that causes acute inflammation by stimulating host cells to produce pro-inflammatory cytokines, has been used to induce in vivo animal models of ALI that closely resemble the symptoms in humans [4], such as diffuse damage to the alveolar epithelium and capillary endothelium [5] and inflammatory cell influx [6,7], and plays a pivotal role in the progression of ALI. Despite the urgent need for ALI therapy, there have been no benefits to patients with ALI owing to low efficacy and severe side effects, such as dexamethasone, a corticosteroid commonly used to manage inflammatory responses in ALI, leading to to other adverse effects such as hyperglycemia, osteoporosis, and adrenal suppression after prolonged administration [8]. Thus, there is an appealing need for treatment innovation in ALI.

The NLRP3 inflammasome is a large intracellular multi-protein complex consisting of NLRP3, ASC, and caspase-1 [9]. The NLRP3 inflammasome plays a central role in innate immunity against stimuli that are associated with infection or cellular stress, causing the activation of caspase-1 to promote the maturation of the pro-inflammatory cytokines interleukin (IL)-1β and IL-18 [10,11,12]. Furthermore, activation of the NLRP3 inflammasome assists the host in resisting the spread of invading bacteria and pathogens, which can lead to inflammation-related tissue damage due to overactivation [13,14]. Recent progress in ALI has verified that the full development of ALI requires the engagement of the NLRP3 inflammasome [15] due to a wide variety of commensal and pathogenic bacteria, viruses, and fungi on the surface of the lung [16]. Moreover, emerging studies have revealed the role of inflammasomes in ALI triggered by infections, such as by *Chlamydia pneumoniae* [17], *Streptococcus pneumoniae* [18,19], influenza A virus [20,21], *Staphylococcus aureus* [20,22], *Mycobacterium tuberculosis* [23], and *Pseudomonas aeruginosa* [24,25]. NLRP3 inflammasome activation in alveolar macrophages (AMs) increases caspase-1 activity and IL-1β production and plays a crucial role in LPS-induced ALI [26]. Thus, targeting NLRP3-inflammasome-associated pathways in ALI is of important research significance.

**Figure 1:**
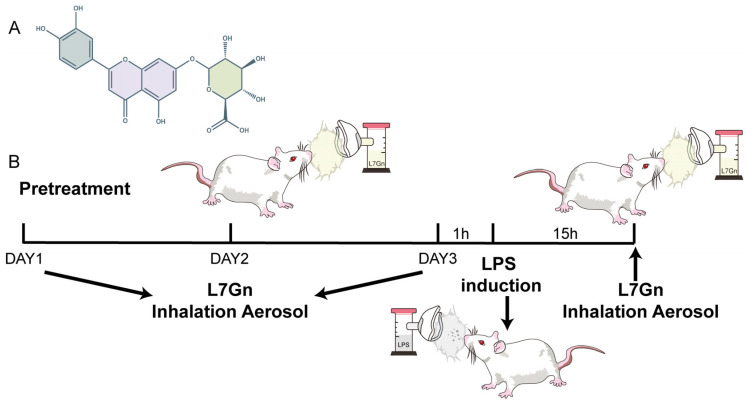
The structure of L7Gn (**A**), the compound of interest, and the experimental design of the study (**B**).

Traditionally, pharmacologically active Chinese herbal medicine extracts used to treat diseases have the same chemical constituents. Luteolin-7-O-glucuronide (L7Gn) is a flavonoid (Figure 1A). Numerous anti-inflammatory herbal medicine extracts have been reported, such as those of *Salvia officinalis* [27], *Ixeris sonchifolia* Hance [28], *Phaeocystis globose* [29], *Enhalus acoroides* [30], *Rosmarinus officinalis* L. [31], *Mentha labiatae* [32], and *Crepidiastrum denticulatum* [33]. Previous research has revealed that L7Gn reduces the levels of inflammatory mediators by inhibiting TAK1, NF-κB, p38, and JNK and upregulates the expression of anti-oxidative regulators by Nrf2, exhibiting anti-inflammatory and anti-oxidative effects [34,35]. Recent progress in the determination of the anti-inflammatory activity of luteolin, the mother compound of L7Gn, has shown that luteolin could significantly protect against NLRP3-mediated inflammatory diseases by inhibiting NLRP3 inflammasome activation [36,37,38,39]. However, the effect of L7Gn on lung inflammation and the underlying mechanisms remain unknown.

In this study, our research aimed to introduce L7Gn into an aerosol inhalation solution to achieve local administration and to evaluate its anti-inflammatory effects in an ALI rat model. In addition, our research attempted to explore the mechanism of inhibition of NLRP3 inflammasomes by L7Gn in MH-S cells, which might provide new treatment options against ALI through inhibiting NLRP3 inflammasome activation.

## 2. Results

### 2.1. Determination of the Real-Time Particle Size Distribution

**Figure 2:**
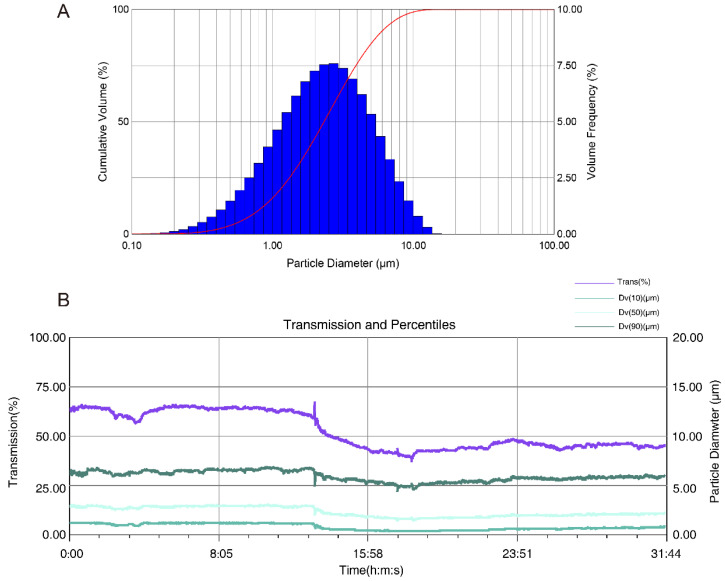
The parameter of real-time particle size of L7Gn. (**A**) The histogram of particles diameter; (**B**) Real-time transmission and percentiles of inhalation of aerosolized L7Gn, Trans (purple line) Dv(10) (dark green line), Dv(50) (light green line), and Dv(90) (green line).

The transmission and size diameters could be detected by the Spraytec spray droplet size measurement system. The higher the transmission was, the lower the particle size diameters were. For deposition during the duration of aerosol inhalation, the inhalation of aerosolized L7Gn could be stably generated using the nebulizer (Figure 2B). Meanwhile, Dv(10), Dv(50), and Dv(90) represent the size values when 10%, 50%, and 90% of the particle population accumulates, respectively, as shown in Figure 2A and Table 1. The average values of Dv(10), Dv(50), and Dv(90) of L7Gn (4 mg/mL) were 0.77, 2.32, and 5.91 μm, respectively. Moreover, Dv(50) is the main parameter of particle size distributions, and the Dv(50) of L7Gn mean value between 1 and 5 µm indicates that 4 mg/mL L7Gn solution could be successfully delivered to the lung for optimal deposition [40].

### 2.2. L7Gn Protects Against Pulmonary Function Damage

**Figure 3:**
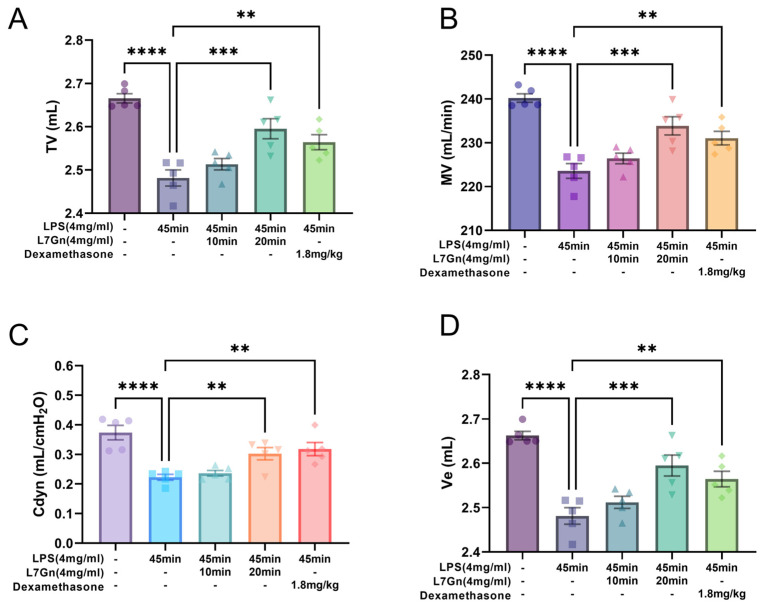
The effect of L7Gn on pulmonary function in LPS-induced ALI rats. Pulmonary function was measured quantitatively by assaying for TV, MV, Cdyn, and Ve (**A**–**D**). All the data presented as mean ± SEM. *n* = 5. **, *p* < 0.01; ***, *p* < 0.001; ****, *p* < 0.0001.

To determine the protective effect of L7Gn against lung injury in LPS-induced ALI rats, lung function tests of tidal volume (TV, mL), minute volume (MV, mL/min), airway resistance (RI, cmH_2_O*s/mL), dynamic compliance (Cdyn, mL/cmH_2_O), and minute ventilation (Ve, mL) were performed. As depicted in Figure 3A–D, compared to those of the normal group, the TV, MV, Cdyn, and Ve of rats were significantly decreased in the LPS-induced ALI group (*p* < 0.0001). However, pretreatment with L7Gn and dexamethasone enhanced the lung function. The TV, MV, Cdyn, and Ve values were significantly increased in the L7Gn 20 min (41.6 mg/kg) and dexamethasone (1.8 mg/kg) groups. These results suggested that aerosol inhalation of L7Gn protected the rats from LPS-induced lung injury.

### 2.3. L7Gn Inhibits LPS-Induced Pulmonary Injury

**Figure 4:**
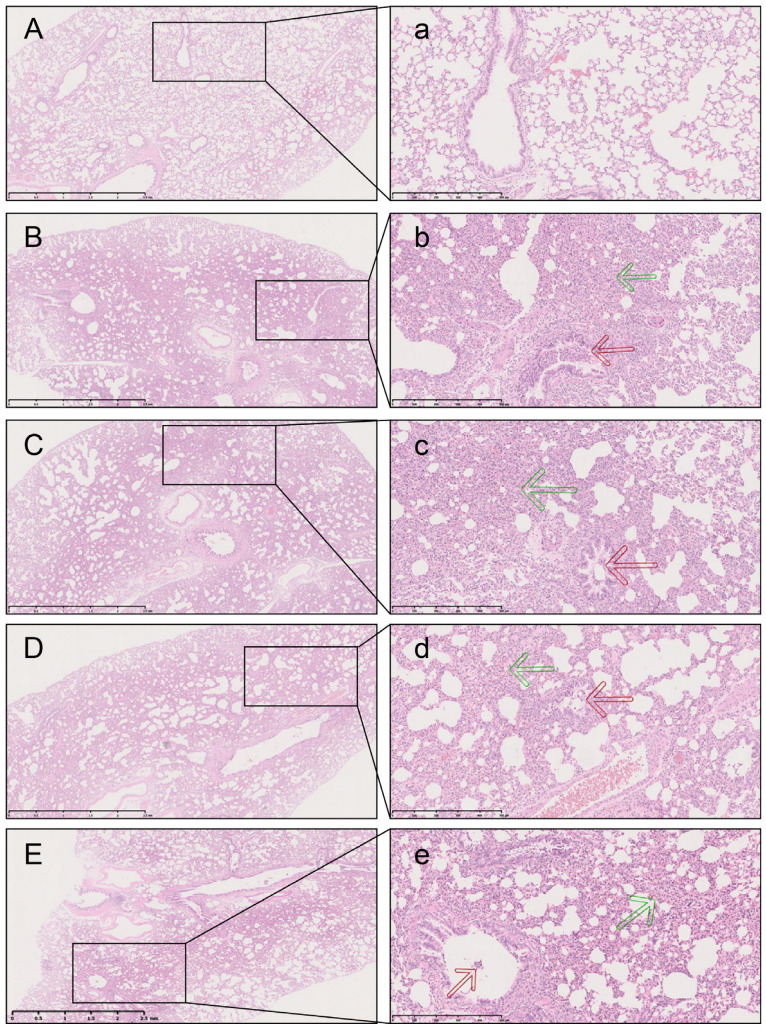
L7Gn inhibits LPS-induced pulmonary damage in rats. Tissue sections of a representative lung section from each group: normal group (**A**,**a**), LPS + vehicle group (**B**,**b**), LPS + L7Gn 10 min group (**C**,**c**), LPS + L7Gn 20 min group (**D**,**d**), and LPS + dexamethasone group (**E**,**e**), which were stained with hematoxylin and eosin (H&E) ((**A**–**E**), 25×; (**a**–**e**), 100×), bronchiolar inflammation (red arrow), and interstitial thickening (green arrow).

The lung tissue in the LPS group was visibly damaged, with hemorrhaging and thickening of the alveolar wall, as shown by the green arrow in Figure 4b. Inflammatory cells infiltrated into the lung parenchyma, as evidenced by an increase in lung injury score (*p* < 0.001). Conversely, the L7Gn and dexamethasone groups showed significantly reduced bronchiolar inflammation (Table 2) and remission of thickening in the alveolar space and lung interstitium and hemorrhage. Together, these results provide important insights regarding the inhibition of LPS-induced lung tissue injury by L7Gn and dexamethasone.

### 2.4. L7Gn Inhibits NLRP3 Inflammasome Activation in LPS-Induced ALI Rats

**Figure 5:**
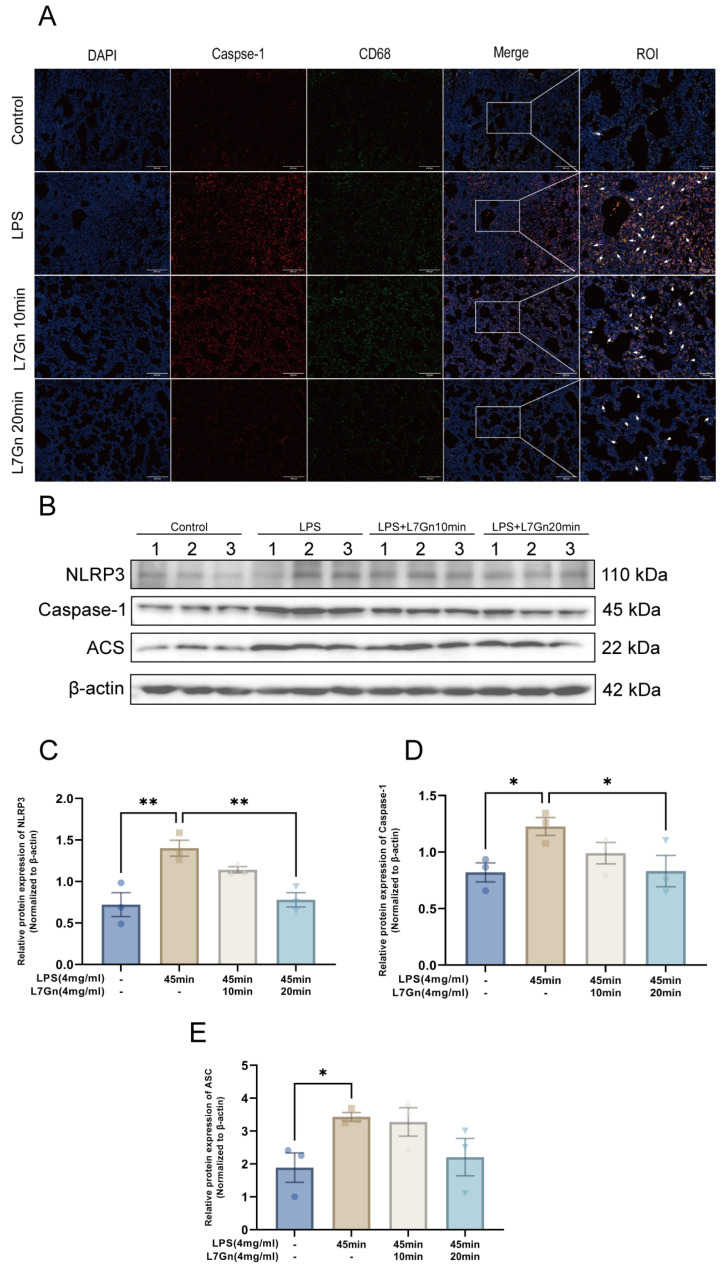
L7Gn inhibited NLRP3 inflammasome activation in LPS-induced rats. Sections of lung tissue were immunostained with CD68 (green) and Caspase-1 (red) antibodies shown as representative images (**A**); Representative Western blotting of NLRP3, Caspase-1, and ASC (**A**): The relative protein expression of NLRP3, Caspase-1, and ASC normalized to that of β-actin. (**B**–**E**). All data are presented as mean ± SEM., *n* = 3. * *p* < 0.05; **, *p* < 0.01.

L7Gn inhibited inflammation by inhibiting the NLRP3 inflammasome, NLRP3 inflammasome activation in alveoli macrophages (AMs) increases caspase-1 activity and IL-1β production, which is confirmed to play a crucial role in LPS-induced ALI [26]. As shown in Figure 5, our research detected the CD68-positive macrophages (green) and Caspase-1 (red) expression in lung tissues by immunofluorescence and the protein expression of NLRP3 using Western blotting. According to the results, L7Gn could reduce the higher number of infiltrative inflammatory immune cells (Figure 5). The CD68-positive macrophages were present in larger numbers in the LPS group; L7Gn could reduce the number of macrophages, while capase-1 has lower expression in the L7Gn groups. Then, L7Gn exerted a protective effect by inhibiting the expression of NLRP3 and caspase-1 in inflamed lung tissue (*p* < 0.01 or *p* < 0.05). Furthermore, L7Gn decreased the expression of ASC, but not significantly. These results suggested that L7Gn suppressed the activation of the NLRP3 inflammasome in lung tissue.

**Figure 6:**
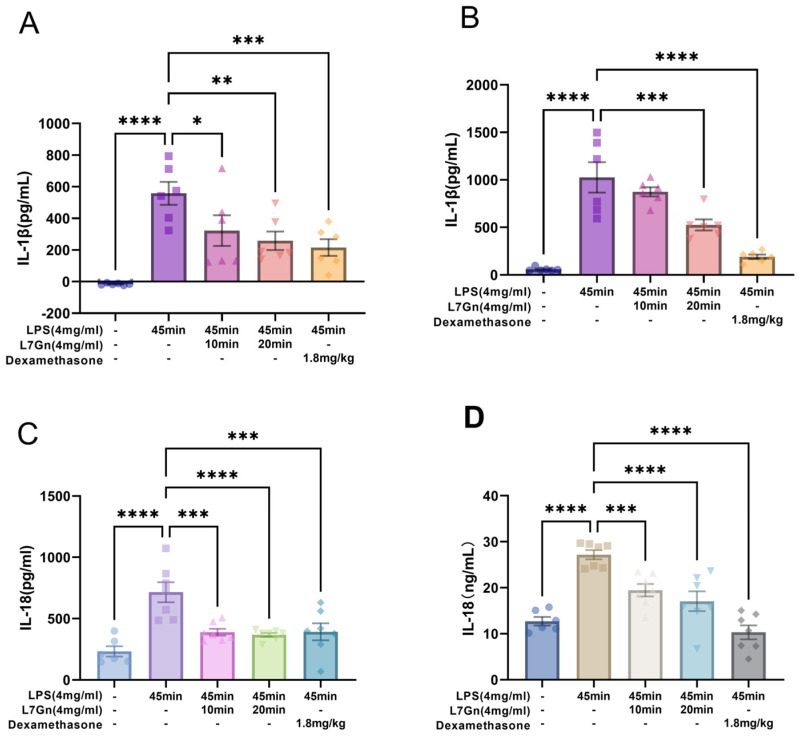
L7Gn decreased the levels of inflammatory cytokines in LPS-induced bronchiolar inflammation. The levels of IL-1β in BALF (**A**) and lung homogenates (**B**) and IL-18 in BALF (**C**) and lung homogenates (**D**) were determined using ELISA kits. All data are presented as mean ± SEM., *n* = 6. * *p* < 0.05; **, *p* < 0.01; ***, *p* < 0.001; ****, *p* < 0.0001.

To further determine whether L7Gn could repress the downstream inflammation of NLRP3 inflammasome activation in vivo, the concentrations of IL-1β and IL-18 were measured in bronchoalveolar lavage fluid (BALF) and lung tissue homogenates, as shown in Figure 6. The results showed that L7Gn reduced the generation of IL-1β and IL-18, suggesting that L7Gn could ameliorate inflammatory symptoms in LPS-induced ALI rats.

### 2.5. L7Gn Inhibits NLRP3 Inflammasome Activation in MH-S Cells Induced by LPS

**Figure 7:**
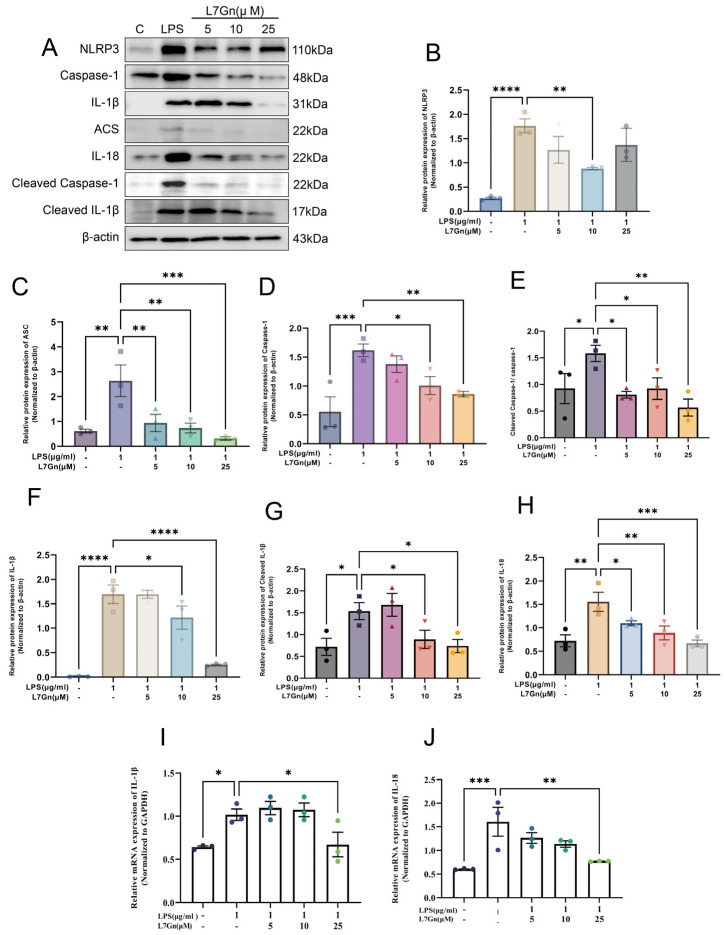
L7Gn inhibited NLRP3 inflammasome activation in LPS-induced MH-S cells. Representative Western blotting of NLRP3, caspase-1, IL-1β, ASC, IL-18, cleaved IL-1β, and cleaved caspase-1 (**A**). The relative protein expression of NLRP3, caspase-1, IL-1β, ASC, IL-18, cleaved IL-1β, and cleaved caspase-1 was normalized to that of β-actin (**B**–**H**). Relative mRNA expression of IL-1β and IL-18 (**I**,**J**). All data are presented as mean ± SEM., *n* = 3. * *p* < 0.05; **, *p* < 0.01; ***, *p* < 0.001; ****, *p* < 0.0001.

To further explore the protective effect of L7Gn via the inhibition of NLRP3 inflammasome activation in vitro, as illustrated in Figure 7, our experiments measured the protein levels of NLRP3, caspase-1, and ASC in MH-S cells, which comprise the NLRP3 inflammasome complex. Notably, NLRP3, ASC, and caspase-1 protein levels increased in the LPS group and were significantly suppressed by L7Gn treatment, as expected (Figure 7B–D). In addition, it was shown that NLRP3 inflammasome activation could induce pro-caspase-1 activation [41]; interestingly, the activation of caspase-1 (cleaved caspase-1) was significantly inhibited by L7Gn (*p* < 0.05 or *p* < 0.01) (Figure 7E).

Based on the activation of caspase-1 to induce the maturation of IL-1β and IL-18, causing inflammation [42], further analysis showed that both the mRNA and protein levels of IL-1β and IL-18 were increased in the LPS group and could be effectively reduced by L7Gn treatment (Figure 7F–J). Overall, these results indicate that the activation of the NLRP3 inflammasome can be inhibited by L7Gn treatment.

## 3. Discussion

Aerosol inhalation is the most straightforward way of administration for the delivery of respiratory agents because the aerosol bypasses first-pass hepatic metabolism and enters into the blood for rapid efficacy [43,44]. Furthermore, inhalation therapy can be administered to reduce adverse effects [45]. Thus, our experiments conclude that there is a viable location for the L7Gn deposition within the respiratory tract owing to the atomization characteristics of the aerosol particles via the generation impactor. Therefore, we present that the drug delivery of L7Gn by inhalation offers a straightforward treatment for ALI to rapidly and efficiently enter the lung. The findings from the Spraytec spray droplet size measurement system reveal that the aerosolized L7Gn solution, with a Dv(50) value of 2.32 µm, resides within the optimal size range for effective pulmonary delivery [46]. This size distribution suggests a promising potential for deep lung penetration, thereby maximizing therapeutic efficacy by ensuring that L7Gn reaches the lower respiratory tract where it can exert its intended effects. The ability to achieve such particle sizes indicates that our formulation approach is well-suited for inhalation therapy, positioning L7Gn as a promising candidate for respiratory treatments. The successful aerosolization and deposition characteristics underscore the feasibility of using nebulization as a delivery method, aligning with our objective to develop efficient and targeted therapies with minimal systemic side effects. Future optimization of formulation and delivery parameters could further enhance the potential of L7Gn in clinical applications.

Inflammation is the response of the body to an injury or threat. LPS has been widely used to induce pulmonary inflammation in ALI animal models, providing for studying the mechanisms of various diseases, discovering new biomarkers, and identifying drug targets [4]. In this study, we confirmed that L7Gn exerted protective effects against LPS-induced inflammation via the aerosol inhalation route by enhancing lung function and reducing lung damage by ameliorating inflammatory cell infiltration in lung tissues. Notably, nebulization of L7Gn may be a more efficient therapeutic strategy for ALI.

Macrophages are innate immune cells that play crucial roles in controlling and regulating immune responses. Previous studies have reported that the activation of NLRP3 inflammasomes in macrophages plays a vital role in ALI progression. Thus, the inhibition of NLRP3 inflammasome activation may be an effective approach for ALI treatment. Once the NLRP3 inflammasome is activated, ASC, a significant adaptor protein for recruiting the inflammasome adaptor protein, is recruited and then interacts with caspase-1, leading to its activation [47]. In this study, the expression of NLRP3 inflammasome proteins such as NLRP3, caspase-1, and ASC was repressed by L7Gn in an LPS-induced ALI rat model. As NLRP3 is a critical component of the NLRP3 inflammasome, our experiments used immunofluorescence to assess whether L7Gn could inhibit NLRP3 expression around the infiltrated macrophages in lung tissues. Our research confirmed that L7Gn could significantly reduce the levels of IL-1β and IL-18 in both BALF and lung tissue homogenates. Moreover, the potent pro-inflammatory cytokines IL-1β and IL-18 are synthesized by macrophages, whose activation follows a unique mechanism involving the activation of caspase-1 by the assembly of the NLRP3 inflammasome [15,45], and can lead to severe inflammatory conditions. Previous research has uncovered that L7Gn diminishes the levels of inflammatory mediators by inhibiting TAK1, NF-κB, p38, and JNK, while concurrently upregulating the expression of antioxidative regulators via Nrf2, thus manifesting both anti-inflammatory and antioxidative effects [34,35]. Recent advancements in elucidating the anti-inflammatory properties of luteolin, the precursor compound of L7Gn, have demonstrated that luteolin can substantially safeguard against NLRP3-mediated inflammatory diseases by inhibiting the activation of the NLRP3 inflammasome [36,37,38,39]. Thus, we determined the mechanism underlying the regulatory effect of L7Gn on the NLRP3 inflammasome in macrophages.

To further clarify the results of our in vivo study, our experiments evaluated the anti-inflammatory effects of L7Gn on LPS-stimulated MH-S cells in vitro. The activation of the NLRP3 inflammasome requires two steps: priming and activation. The first priming signal is often induced by toll-like receptor agonists, such as LPS, which upregulates NLRP3, IL-1β, and IL-18 expression. Meanwhile, the “signal two activations” consist of a broad spectrum of infection and stress-associated signals. Once activated, caspase-1 promotes the maturation of IL-1β and IL-18 [10,12]. Additionally, our results showed that the expression of the multi-protein complex (NLRP3–ASC–caspase-1 assembly), as well as the activation ratio of caspase-1 (cleaved form to pro-enzyme form), was significantly diminished by L7Gn. Meanwhile, reverse transcription-polymerase chain reaction (RT-PCR) analysis revealed that L7Gn suppressed the mRNA expression of IL-1β and IL-18 in macrophages. Thus, our in vitro study indicated that L7Gn could inhibit NLRP3 inflammasome priming and activation.

While our study offers valuable insights into the inhibitory effects of L7Gn on the NLRP3 inflammasome pathway, several limitations must be addressed in future research to better elucidate the specific regulatory mechanisms of L7Gn. Firstly, the precise molecular targets of L7Gn within the NLRP3 inflammasome signaling cascade remain unclear. Further studies utilizing techniques such as co-immunoprecipitation and mass spectrometry could aid in identifying potential binding partners or direct targets of L7Gn. Additionally, although our RT-PCR analysis indicated a suppression of IL-1β and IL-18 mRNA expression, it would be advantageous to investigate whether L7Gn influences other upstream transcription factors or signaling pathways involved in inflammasome regulation. Moreover, the long-term effects and potential off-target consequences of L7Gn should be evaluated to ensure its safety and efficacy as a therapeutic agent. Addressing these aspects will provide a more comprehensive understanding of L7Gn’s mechanism of action and its potential application in the treatment of inflammatory diseases.

## 4. Materials and Methods

### 4.1. Chemicals and Reagents

Murine alveolar macrophage cell line (MH-S) was obtained from ATCC (Manassas, VA, USA). Luteolin-7-O-glucuronide (L7Gn, CAS:29741-10-4) was acquired from Chengdu Herbpurify Co., Ltd. (Chengdu, Sichuan, China) and stored in a refrigerator at −20 °C, frozen, and in the dark, until use when preparing the inhalation solution. Dexamethasone sodium phosphate was purchased from Guizhou Tiandi Pharmaceutical Co. Ltd. (Guizhou, China). LPS from *Escherichia coli* O55:B5(L2880) was acquired from Sigma-Aldrich (Darmstadt, Germany). NLRP3 antibody (ab263899) was obtained from Abcam (Cambridge, UK), and caspase-1 antibody (sc-134306) and ASC antibody (sc-514414) from Santa Cruz Biotechnology (Santa Cruz, CA, USA). The triple fluorescent staining kit was obtained from Beijing Xuebang Technology Co, Ltd. (Beijing, China).The Mouse Reactive Inflammasome Antibody Sampler Kit (#20836) was obtained from Cell Signaling Technology (Danvers, MA, USA). The IL-1β and IL-18 ELISA kits were purchased from Beijing Solarbio Science & Technology Co., Ltd. (Beijing, China).

### 4.2. Dose and Method of Administration

The solution of L7Gn was dissolved in 0.01 M phosphate-buffered saline (PBS) to a concentration of 4 mg/mL, and the rats were administered with the atomized solution by inhalation NIES (Melton Lab Co., LTD Lab, Shanghai, China). The administration of the aerosolized L7Gn solution was performed using a Pari Boy SX nebulizer, paired with the Melton Inhalogic NIES nose-only exposure tower. The actual dose was estimated based on established parameters for inhalation exposure, ensuring precise and consistent delivery. The actual atomization dose was calculated according to the formula: *Dose = RMV* × *C* × *D* × *F*/*BW* [48]. The two groups were atomized for 10 and 20 min. The actual doses were 20.8 mg/kg and 41.6 mg/kg.

### 4.3. Determination of Real-Time Particle Size for Aerosol Inhalation

A generation pump (Pari Pharma GmbH, Starnberg, Germany) and Pari Boy SX nebulizer with the red nozzle insert were used to generate the L7Gn aerosols. The particle size diameters and transmission of aerosols of L7Gn were determined using a Spraytec spray droplet size measurement system (Malvern, Worcestershire, UK), which generated D10, D50, and D90 values relating to mean maximal particle size diameters, representing 10%, 50%, and 90% of particles, respectively.

### 4.4. Animal Experimental Design

Male specific pathogen free-grade Wistar rats with body weights of approximately 220 g from Beijing Vital River Laboratory Animal Technology Co., Ltd.(Beijing, China), were raised under a 12 h light/dark cycle for one week to acclimatize them to the new conditions.

ALI was induced by the inhalation of aerosolized LPS [49]. The Melton Inhalogic NIES nose-only exposure tower was employed for the administration of aerosolized LPS and treatments. The animals were divided into five groups (*n* = 10): normal, LPS, LPS + L7Gn 10 min, LPS + L7Gn 20 min, and LPS + dexamethasone. The rats were subjected to inhalation of aerosolized L7Gn in the LPS + L7Gn group and intraperitoneally injected with dexamethasone (5 mg/kg) in the LPS + dexamethasone group daily for three days before ALI induction. LPS-induced ALI was stimulated by simultaneous exposure to 4 mg/mL aerosolized LPS for 45 min. The control group received aerosolized sterile saline. Finally, another dose of L7Gn or dexamethasone was administered 15 h after LPS exposure. After one hour, the rats were anesthetized by isoflurane inhalation for lung function experiments (Figure 1B).

### 4.5. Determination of the Lung Function

The key parameters related to the pulmonary function of the experimental rats were determined in real-time after the rats were anesthetized using an RC system (DSI, Delaware, MN, USA), including tidal volume (TV, mL), minute volume (MV, mL/min), airway resistance (RI, cmH_2_O*s/mL), dynamic compliance (Cdyn, mL/cmH_2_O), and minute ventilation (Ve, mL). 

### 4.6. BALF Collection

BALF was harvested by injecting 2 mL PBS into the lungs three times. The collected BALF was then centrifuged for 7 min at 400× *g* and 4 °C. The supernatant was stored at −80 °C for enzyme-linked immunosorbent assay (ELISA) detection.

### 4.7. ELISA of BALF and Lung Homogenates

Lung tissues (100 mg) were snap-frozen and later processed to obtain lung homogenates. The protein concentration of the lung homogenates was determined using the BCA method (Pierce BCA kit, Thermo Fisher Scientific, Waltham, MA, USA). According to the manufacturers’ instructions, the Solarbio kits were used to measure the IL-1β and IL-18 levels of BALF and lung homogenates. The optical density was read using a Spectramax i3x reader (Molecular Devices, San Jose, CA, USA) at 450 nm.

### 4.8. Histological Analysis and Immunofluorescence

The right lung was removed and fixed in 4% paraformaldehyde. Lung tissue sections were then stained with hematoxylin and eosin. The degree of inflammation in the bronchi and bronchioles was determined according to a previously reported method with slight modifications [50]. Grade 1 indicated that the degree of the lesion was slight (<10%), grade 2 indicated that the degree of the lesion was mild (10–30%), grade 3 indicated that the degree of the lesion was moderate (31–50%), and grade 4 indicated that the degree of the lesion was severe (>50%). Images were acquired using a digital sight DS-Fi2 camera (Nikon, Tokyo, Japan).

Fluorescent staining was carried out using a TSAPLus triple fluorescent staining kit following the manufacturer’s instructions. The primary antibodies, including Caspase-1 (1:50) and CD68 (1:200), were incubated in lung slices, respectively, overnight at 4 °C; the secondary antibody (HRP-labelled goat anti-rabbit IgG, 1:250)was added and incubated at 37 °C for 20 min. Next, TSA-555 and TSA-647 working solutions were incubated for 10 min at 25 °C in the dark. Nuclei were counterstained with DAPI. Representative immunofluorescence images were visualized by Pannoramic MIDI and Pannoramic Scanner (3D Histech Ltd., Budapest, Hungary).

### 4.9. Inflammasome Activation Assays in MH-S Cells

The MH-S cell line was cultured in Roswell Park Memorial Institute medium with 10% fetal bovine serum and 0.05 mM 2-mercaptoethanol. The cells were plated in a six-well plate (1 × 10^6^ cells/well) and co-incubated with 5 mΜ, 10 μM, and 25 μM L7Gn and LPS (1 μg/mL) for 15 h. The cells were then collected for RT-PCR and Western blot analysis.

### 4.10. RNA Isolation and Quantitative RT-PCR

Total RNA was extracted from MH-S cells using the RNA Easy Fast Cell kit (TIANGEN, Beijing, China). RNA was quantified using a NanoDrop Spectrophotometer (NanoDrop Technologies, Wilmington, DE, USA). RNA was reverse-transcribed into cDNA using *EasyScript* First-Strand cDNA Synthesized SuperMix (TRAN, Beijing, China) according to the manufacturer’s instructions. Target gene fragments were amplified using the TransStar Top Green qPCR SuperMix (TRAN) and CFX96 Real-Time PCR detection system (Bio-Rad, ChemiDoc MP, Bio-Rad, Hercules, CA, USA) according to the manufacturers’ instructions. Gene expression was quantified using the ∆∆Cq method in the CFX Manager Software 1.1 (Bio-Rad, ChemiDoc MP, Bio-Rad) and normalized to GAPDH expression. Specific primer sequences were synthesized by Biomed Company (Beijing, China) and are shown in Table 3. The experiments were performed in triplicate.

### 4.11. Western Blotting

The lung tissues and MH-S cells were lysed with lysis buffer containing protease for protein extraction (CWBIO, Beijing, China). Electrophoresis was performed on the gels using 7.5% and 12.5% SDS-PAGE gel fast preparation kits (Biotides, Beijing, China), and the proteins were transferred to PVDF membranes. The PVDF membranes carrying the proteins were blocked with 5% nonfat milk for 1 h at 25 °C and then incubated with primary antibodies against NLRP3, Caspase-1, IL-1β, ASC, IL-18, cleaved IL-1β, and cleaved caspase-1 overnight at 4 °C and with secondary antibodies for 1 h at 25 °C. Then, Stripping Buffer (CWBIO, Beijing, China) was used for the recycling of deproteinized membrane in our experiment. Finally, immunoblots were visualized using Amersham ImageQuant 800 (GE Healthcare, Buckinghamshire, UK). The quantitative density of the membranes was analyzed using the ImageJ software (ImageJ 1.475v, NIH).

### 4.12. Statistical Analysis

Statistical analysis was performed using SPSS version 23.0 (SPSPSS Inc., Chicago, IL, USA). The difference in the degree of bronchiolar inflammation was assessed using the Kruskal–Wallis rank sum test followed by a post hoc Dunns Kruskal–Wallis multiple comparison test. The remaining comparative studies used a one-way ANOVA followed by LSD post hoc tests. These data are referred to as mean S.E.M. expressed. (standard error of the mean).

## 5. Conclusions

In conclusion, this study demonstrated that L7Gn was prepared as an aerosol inhalation solution and administered into the lungs to exert an anti-inflammatory effect in an LPS-induced ALI rat model. In addition, L7Gn suppressed LPS-induced NLRP3 inflammasome activation in AMs. These findings suggest that aerosol inhalation of luteolin-7-O-glucuronide exerts anti-inflammatory effects by inhibiting NLRP3 inflammasome activation, which provides credible evidence for luteolin-7-O-glucuronide as a potential bioactive chemical against NLRP3 inflammasome activation-related inflammatory diseases.

## Figures and Tables

**Table 1:** Characterization of Aerosolized L7Gn: Transmission Percentage and Volume Distribution (Dv10, Dv50, Dv90).

Title	Average	б	Min	Max
Trans (%)	51.70	9.06	37.50	66.80
Dv(10) (μm)	0.77	0.31	0.36	1.27
Dv(50) (μm)	2.32	0.48	1.48	3.04
Dv(90) (μm)	5.91	0.52	4.47	6.88

**Table 2:** Assessment of Bronchiolar Inflammation (*n* = 10).

Group	Bronchiolar Inflammation Grade					*p*-Value
	0	1	2	3	4	
Control	10	0	0	0	0	
LPS	0	2	7	1	0	0.0001 ^##^
LPS + L7Gn	0	8	2	0	0	0.044 *
LPS + L7Gn	0	9	1	0	0	0.020 *
LPS + Dex	1	6	3	0	0	0.046 *

^##^ *p* < 0.01 vs. the normal group; * *p* < 0.05 vs. the LPS group.

**Table 3:** Primer Sequences Utilized in Real-Time PCR Analyses.

Gene	Primer Forward (5′ → 3′)	Primer Reverse (5′ → 3′)
*IL-1β*	TGTTCTTACAGGAGAGGGTAGAC	GTTCATCTCGGAGCCTGTAGTG
*IL-18*	GACAGCCTGTGTTCGAGGATATG	TGTTCTTACAGGAGAGGGTAGAC
*GADPH*	GTTCATCTCGGAGCCTGTAGTG	ATGCCAGTGAGCTTCCCGTTCAG

## Data Availability

The data that support the findings of this study are available on request from the corresponding author, Guangping Zhang. The data are not publicly available due to privacy concerns.
